# Age-Specific Cut-off Values of Amino Acids and Acylcarnitines for Diagnosis of Inborn Errors of Metabolism Using Liquid Chromatography Tandem Mass Spectrometry

**DOI:** 10.1155/2019/3460902

**Published:** 2019-01-06

**Authors:** Suprovath Kumar Sarker, Md Tarikul Islam, Aparna Biswas, Golam Sarower Bhuyan, Rosy Sultana, Nusrat Sultana, Shagoofa Rakhshanda, Mst. Noorjahan Begum, Asifuzzaman Rahat, Sharmina Yeasmin, Mowshori Khanam, Asim Kumar Saha, Farjana Akther Noor, Abu A. Sajib, Abul B. M. M. K. Islam, Syeda Kashfi Qadri, Mohammod Shahidullah, Mohammad Abdul Mannan, A. K. M. Muraduzzaman, Tahmina Shirin, Sheikh Maksudur Rahman, Syed Saleheen Qadri, Narayan Saha, Sharif Akhteruzzaman, Firdausi Qadri, Kaiissar Mannoor

**Affiliations:** ^1^Laboratory of Genetics and Genomics, Institute for Developing Science and Health Initiatives, Mohakhali, Dhaka 1212, Bangladesh; ^2^Department of Genetic Engineering & Biotechnology, University of Dhaka, Dhaka 1000, Bangladesh; ^3^Infectious Diseases Laboratory, Institute for Developing Science and Health Initiatives, Mohakhali, Dhaka 1212, Bangladesh; ^4^Bangladesh University of Health Sciences, Bangladesh; ^5^Department of Virology, Dhaka Medical College, Dhaka 1000, Bangladesh; ^6^Department of Gynecology & Obstetrics, Bangladesh Institute of Health Sciences General Hospital, 125/1, Darus Salam, Mirpur-1, Dhaka 1216, Bangladesh; ^7^Ain Shams General Hospital and Diagnostic Centre Pvt. Ltd., Vhushir Mill, Sign Board, Gazipur, Bangladesh; ^8^Narsingdi Sadar Hospital, Raipur, Narsingdi, Bangladesh; ^9^Department of Biochemistry and Molecular Biology, University of Dhaka, Bangladesh; ^10^Department of Paediatric Medicine, KK Women's and Children's Hospital, Singapore; ^11^Department of Neonatology, Bangabandhu Sheikh Mujib Medical University, Shahbag, Dhaka, Bangladesh; ^12^Department of Virology, Institute of Epidemiology, Disease Control and Research, Mohakhali, Dhaka, Bangladesh; ^13^Research and Development, Incepta chemicals Ltd., Barabaria, Saturia, Dhankora, Dhaka 1810, Bangladesh; ^14^Pediatric Neurology, National Institute of Neurosciences & Hospital, Dhaka 1207, Bangladesh; ^15^Department of Enteric and Respiratory Infectious Diseases, Infectious Diseases Division, International Centre for Diarrhoeal Disease Research, Bangladesh, Mohakhali, Dhaka, Bangladesh

## Abstract

Liquid Chromatography tandem mass spectrometry (LC-MS/MS) is used for the diagnosis of more than 30 inborn errors of metabolisms (IEMs). Accurate and reliable diagnosis of IEMs by quantifying amino acids (AAs) and acylcarnitines (ACs) using LC-MS/MS systems depend on the establishment of age-specific cut-offs of the analytes. This study aimed to (1) determine the age-specific cut-off values of AAs and ACs in Bangladesh and (2) validate the LC-MS/MS method for diagnosis of the patients with IEMs. A total of 570 enrolled healthy participants were divided into 3 age groups, namely, (1) newborns (1-7 days), (2) 8 days–7 years, and (3) 8–17 years, to establish the age-specific cut-offs for AAs and ACs. Also, 273 suspected patients with IEMs were enrolled to evaluate the reliability of the established cut-off values. Quantitation of AAs and ACs was performed on an automated LC-MS/MS system using dried blood spot (DBS) cards. Then the specimens of the enrolled clinically suspected patients were analyzed by the established method. Nine patients came out as screening positive for different IEMs, including two borderline positive cases of medium-chain acyl-CoA dehydrogenase deficiency (MCAD). A second-tier test for confirmation of the screening positive cases was conducted by urinary metabolic profiling using gas chromatography- mass spectrometry (GC-MS). Out of 9 cases that came out as screening positive by LC-MS/MS, seven cases were confirmed by urinary GC-MS analysis including 3 cases with phenylketonuria, 1 with citrullinemia type II, 1 with methylmalonic acidemia, 1 with isovaleric acidemia and 1 with carnitine uptake defect. Two borderline positive cases with MCAD were found negative by urinary GC-MS analysis. In conclusion, along with establishment of a validated LC-MS/MS method for quantitation of AAs and ACs from the DBS cards, the study also demonstrates the presence of predominantly available IEMs in Bangladesh.

## 1. Introduction

Inborn errors of metabolism (IEMs) are a group of phenotypically and genotypically heterogeneous metabolic disorders caused by mutations in the genes that encode enzymes of the metabolic pathways [1]. Deficiency or altered activities of the necessary enzymes or proteins in the intermediary metabolic pathways leads to a wide spectrum of diseases with clinical heterogeneity [2, 3]. Although, individually these disorders are rare, collectively they are numerous and the prevalence rate varies between 1 in 800 and 1 in 2500 live births [1, 4–6]. Moreover, there is population-wise variation of incidence of IEMs and it is assumed that 6-8% of world population can be affected by these disorders.

Till date, about 1000 IEMs disorders have been identified [7]. However, the clinical manifestations of these disorders are often nonspecific and sometimes the signs and symptoms of an individual IEM overlap with other IEMs and even non-IEMs diseases, such as septicemia [2, 8, 9]. Remarkably, one-third of the IEMs are characterized by involvement of the nervous system [10] and the repercussions of neurometabolic changes, particularly to neurological development during the early years of life make it imperative to detect and treat them at the earliest to prevent forthcoming disaster. The quality of life of the affected individuals can be improved by early diagnosis followed by immediate treatment initiation, provided that treatments are available. Also, preventive measures can be taken through genetic counseling [10]. Many IEMs including aminoacidopathies, organic acidemias, and fatty acid oxidation (FAO) disorders can be treated by simple diet therapy or other easy interventions [11, 12]. Unfortunately, diagnosis of individual IEMs are burdensome and practically impossible, because there is no single test available to diagnose these disorders.

Currently, liquid chromatography-tandem mass spectrometry (LC-MS/MS)-based high throughput technologies are being widely used which permits simultaneous quantification of several metabolites such as amino acids and acylcarnitines from a very small amount of biological specimen. However, age-specific reference ranges of cut-off values for each analyte must be established first for each population by individual laboratories prior to screening/diagnosis of the patients [13–16], because the cut-offs are highly influenced by various factors such as genetic background, geographical location of a population, diet and age [13, 17, 18]. At present, newborns are being screened or diagnosed for more than 30 IEMs using LC-MS/MS through newborn screening programs in the majority of the developed countries, and certain developing countries across the globe [1, 10, 19–22]. Unfortunately, newborn screening facilities do not exist in Bangladesh. As a result, biological specimens from individuals with suspected IEMs are being sent to other countries for screening purposes. However, the approach to analyze specimens in other countries is unaffordable for most Bangladeshis, and above all, the results are interpreted by comparing the data with the cut-off values derived from the people of other geographical location and genetic background.

In this study, we validated the method of LC-MS/MS-based quantification of amino acids and acylcarnitines with the target to establish reference values of the metabolites for three different age groups of our population. Finally, we screened clinical specimens and investigated whether the established reference values of amino acids and acylcarnitines could be used to identify the IEMs, especially aminoacidopathies, organic acidemias, and FAO disorders, among the clinically suspected patients with IEMs in Bangladesh. Our study is expected to motivate Bangladeshi health policymakers to initiate a nationwide newborn screening program without further delay to contribute towards fulfilling the SDG goal 3 in time.

## 2. Methods and Materials

### 2.1. Ethical Approval and Study Population

The study protocol was reviewed and approved by the National Ethics Review Committee (NERC) of Bangladesh Medical Research Council (BMRC), Dhaka, Bangladesh (BMRC/NREC/2013-2016/990). Prior to the collection of blood specimens, written consent was obtained from the parents of the patients with IEMs and healthy participants. For the establishment of cut-off values of amino acids and acylcarnitines, a total of 570 healthy participants without any disease were enrolled in the study. The healthy participants were divided into 3 groups according to age, namely, group A (N= 120, aged 1-7 days), group B (N= 243, aged 8 days-7 years), and group C (N= 207, aged 8-17 years). Additionally, 273 patients (158 males and 115 females) with clinical manifestations suggestive of IEMs were also enrolled. The median age of the patients with suspected IEMs was 2.16 years (range: 0.03-17 years). Inclusion criteria for participation in the study were lethargy, irritation, poor feeding, tachypnea, seizures, persistent vomiting, toe-walking, unexplained developmental delay, abnormal movement, language retardation, history of death of previous sibling due to unexplained cause, positive family history with metabolic disorders, and parental consanguinity. In addition, these patients were also subjected to investigation for the signs, metabolic acidosis with an increase in anion gap, persistent or recurrent hypoglycemia, hypotonia, hyperammonemia, splenomegaly, abnormal imaging and electrophysiologic findings, which are suggestive of metabolic disorders [6, 8, 10, 23]. However, the patients with aforementioned symptoms who had history of perinatal brain injury, infection of central nervous system, or chromosomal abnormalities were excluded from the study [10, 23]. Based on the inclusion criteria, the patients with suspected IEMs were referred by the pediatric clinicians from (1) National Institute of Neurosciences & Hospital, Bangladesh, (2) Bangabandhu Sheikh Mujib Medical University, Bangladesh, and (3) Dhaka Shishu Hospital, Bangladesh. All the patients were examined by expert clinicians before enrollment in the study. Before enrollment of the participants, written informed consent was taken from the parents or legal guardians.

### 2.2. Specimen Collection and Storage

The whole blood specimens of newborns were collected by heel prick method and ~75 *μ*L of blood was spotted on Whatman™ 903 Generic Multipart filter paper (GE Healthcare, Westborough, MA, USA) to prepare a DBS (Dried blood spot) card for LC-MS/MS analysis. Approximately, 80% specimens were collected between 24 hours and 72 hours after birth and 20% specimens were collected between day 4 and day 7 after birth. The whole blood specimens for older children were collected after 4-hour fasting using standard venipuncture method and a DBS card was prepared by spotting ~75 *μ*L blood on Whatman™ 903 filter paper. In addition, 5 mL fasting urine specimens were also collected for urinary metabolic screening tests including ferric chloride test, 2,4-Dinitrophenylhydrazine test, Cyanide nitroprusside test, and tests for urine reducing sugar and ketone bodies. The DBS cards were dried for 4 hours at room temperature and stored at -70°C in plastic ziplock bags with desiccants until analysis was done. After urinary metabolic screening tests, the leftover urine specimens (~ 2 mL) were stored at -70°C for second-tier tests such as metabolic profiling using gas chromatography-mass spectrometry (GC-MS).

### 2.3. Specimen Preparation and LC-MS/MS Analysis

A NeoMass AAAC kit (Labsystems Diagnostics Oy, Finland) was used for quantitation of amino acids and acylcarnitines from the DBS cards. A vial of lyophilized isotope-labeled internal standards (IS) containing ^2^H_4_-Alanine (Ala IS), ^2^H_4_-^13^C-Arginine (Arg IS), ^2^H_2_- Citrulline (Cit IS), ^2^H_3_- Leucine (Leu IS), ^13^C_6_-^15^N_2_- Lysine (Lys IS), ^2^H_3_- Methionine (Met IS), ^2^H_6_-Ornithine (Orn IS), ^13^C_6_- Phenylalanine (Phe IS), ^2^H_5_- Proline (Pro IS), ^13^C_3_-Serine (Ser IS), ^13^C_6_- Tyrosine (Tyr IS), and ^2^H_8_- Valine (Val IS), ^2^H_9_- free carnitine (C0 IS), ^2^H_3_- Acetylcarnitine (C2 IS), ^2^H_3_- Propionylcarnitine (C3 IS), ^2^H_3_- Butyrylcarnitine (C4 IS), ^2^H_9_- Isovalerylcarnitine (C5 IS), ^2^H_3_- Glutarylcarnitine (C5DC IS), ^2^H_3_- Hexanoylcarnitine (C6 IS), ^2^H_3_- Octanoylcarnitine (C8 IS), ^2^H_3_- Decanoylcarnitine (C10 IS), ^2^H_3_- Lauroylcarnitine (C12 IS), ^2^H_9_^−^ Myristoylcarnitine (C14 IS), ^2^H_3_- Palmitoylcarnitine (C16 IS), and ^2^H_3_- Stearoylcarnitin (C18 IS) was reconstituted by adding 1 mL of extraction solution which was provided with the NeoMass kit. Daily working extraction solution was prepared by diluting the reconstituted internal standards with 1:100 (v/v) extraction solution.

For analysis of amino acids and acylcarnitines from the stored specimens, the DBS cards were brought to room temperature (+18 to +25°C) prior to extraction and a 3.2 mm disk (equivalent to ~3.1 *μ*L whole blood) was punched out using an automated Wallac Delfia DBS puncher (Perkin-Elmer Life Sciences, Inc., USA) into a well of polystyrene U-bottomed 96-well microplate provided with the kit. After addition of 100 *μ*L daily working extraction solution to each well of microplate, the plate was covered with adhesive film and incubated for 20 minutes at room temperature in a microplate shaker with shaking speed of 650 rpm. After incubation, 70 *μ*L supernatant was transferred into a V-bottomed microplate and covered with aluminum foil to reduce the evaporation. The plate was placed in the autosampler of LC-MS/MS system and 5 *μ*L supernatant was injected into the LC-MS/MS system for analysis.

### 2.4. Instrumentation and LC-MS/MS Analysis

Shimadzu LCMS-8050 liquid chromatograph mass spectrometer (Shimadzu Corporation, Japan) equipped with binary pump, autosampler and electrospray ionization (ESI) source was used for the analysis. The specimens were injected into the LC-MS/MS system through ESI source for atmospheric pressure ionization and specimen analysis was performed using flow injection analysis-electrospray ionization-tandem mass spectrometry (FIA-ESI-MS/MS) method.

### 2.5. Data Acquisition and Data Processing

The solvent delivery pump of LC-MS/MS system was programed for delivery of mobile phase (provided with the kit) at a constant flow rate of 150 *μ*L/min and the data acquisition was done in positive ion multiple reaction monitoring (MRM) mode. The ionization source parameters of LC-MS/MS were interface voltage 4.5 kV, interface temperature 250°C, dissolution line temperature 250°C, heat block temperature 400°C, nebulizing gas flow 3.0 L/min, and drying gas flow 15.0 L/min. Argon was used as a collision gas at pressure of 230 kPa. A Lab Solution (version 5.82 SP1, Shimadzu Corporation, Japan) software was used for data acquisition and the concentration of each analyte was calculated using a Neonatal Solution software (version 2.20, Shimadzu Corporation, Japan). The total run time for each specimen was 1.5 min and the data were acquired for 0.9 min. The MRM parameters for analysis of amino acids and acylcarnitines have been shown in Supplementary Table S1 (Supplementary File 1). Three levels of quality control (QC) specimens (low, medium, and high) were provided with the NeoMass AAAC kit to ensure the accuracy of the test results. These QC specimens were extracted and analyzed parallel with the specimens of healthy controls and patients to monitor the reliability of the data generated from the LC-MS/MS analysis.

### 2.6. Validation of the Method

Validation of the method and performance of the Shimadzu LCMS 8050 (Shimadzu Corporation, Kyoto, Japan) were done using three levels of control specimens (low, medium, and high) provided with the NeoMass AAAC kit (Labsystems Diagnostics Oy, Vantaa, Finland). Extraction of the analytes was done using aforementioned extraction method for the DBS cards and performance of the method was evaluated in terms of intra-assay and inter-assay accuracy and precision, linearity, limit of detection (LOD) or functional sensitivity, limit of quantitation (LOQ), and recovery. The method validation analysis was done for Ala, Arg, Cit, Leu, Lys, Met, Orn, Phe, Pro, Ser, Tyr, Val, C0, C2, C3, C4, C5, C6, C8, C10, C12, C14, C16, and C18.

### 2.7. GC-MS Analysis for Urinary Metabolic Profiling

For the second-tier test, urine specimens (~ 2 mL) of the patients who were screening positive by LC-MS/MS analysis were sent to NeoCare Diagnostics Pvt. Ltd., Mumbai, India, for urinary metabolic screening test using GC-MS.

### 2.8. Data Acquisition Statistical Analysis

GraphPad Prism 7 software (GraphPad Software, Inc., USA) and IBM SPSS Statistics (Version 20) were used for statistical analysis. Percentiles, mean values, standard deviations (SD), coefficients of variation (CV) and relative errors (RE %) were calculated using standard statistical formulas.

## 3. Results

### 3.1. Validation of LC-MS/MS Method for Quantitation of Amino Acids and Acylcarnitines

Intra-assay and inter-assay variability of the method for each analyte were done by analyzing three levels of control specimens in 15 different replicates over a period of 5 days according to the CLSI EP5-A2; Evaluation of Precision Performance of Quantitative Measurement Methods; Approved Guideline—Second Edition [24]. Supplementary Table S2 (Supplementary File 1) shows that the average range of intra-assay percentage of coefficient of variation (%CV) of 11 amino acids (Ala, Arg, Cit, Leu, Lys, Met, Orn, Phe, Pro, Ser, and Val) was within 20% of the target value which was within the acceptable limit [25, 26]. However, the intra-assay %CV for Tyr was 23.15%. The average range of %CV for free carnitine (C0) and acylcarnitines (C0, C2, C3, C4, C5, C8, C10, C12, C14, C16, and C18) was also within 20% of the target value, except for C6 (36.47%) which may have been due to very low concentrations of the analyte in the low level DBS controls which were supplied with the kit (0.13 *μ*mol/L). The accuracy of the method was determined as percentage of relative error (RE%). In case of intra-assay accuracy, the average ranges of RE% of amino acids and acylcarnitines were -19.85 to +9.33% and -6.46 to +6.93%, respectively, which were within the acceptable ranges (Supplementary File 1: Supplementary Table S2) [26]. The average ranges of inter-assay %CV were 1.32%–11.60% and 1.16–14.14% for amino acids and acylcarnitines, respectively. The ranges of average inter-assay RE% were -19.31 to +3.55 % and -6.61 to +8.36% for amino acids and carnitine-acylcarnitines, respectively. These data indicated that the inter-assay accuracy and precision were within acceptable limits (Supplementary File 1: Supplementary Table S3) [25, 26]. The ranges of average recovery rates for amino acids and carnitine-acylcarnitines were 80.68–103.54% and 93.37–108.35%, respectively (Supplementary File 1: Supplementary Table S4) and they were within acceptable limits [26]. Linearity of the method for quantitation of each analyte was evaluated following CLSI EP06; Evaluation of the Linearity of Quantitative Measurement Procedures: A Statistical Approach; Approved Guideline [27]. As shown in Supplementary Table S4 (Supplementary File 1), the coefficient of determination (R^2^ > 0.99) along with slope and y-intersect indicated that the method was linear for all concentration levels (low, mid, and high) of each analyte. LOD and LOQ for all analytes were also calculated following CLSI EP17; Evaluation of Detection Capability for Clinical Laboratory Measurement Procedures; Approved Guideline, and the results have been demonstrated in Supplementary File 1 (Supplementary Table S4) [28]. Together, the data generated for method validation study indicated that this method was suitable for quantitation of amino acids and acylcarnitines from the DBS specimens [25, 26].

### 3.2. Determination of Cut-off Values of Amino Acids, Acylcarnitines, and Marker Ratios

In this study, we calculated the cut-off values using percentile distribution of metabolites in healthy subjects according to CLSI C28-A2; How to Define and Determine Reference Intervals in the Clinical Laboratory; Approved Guideline—Second Edition [29]. The blood concentrations of metabolites (12 amino acids, free carnitine and 23 acylcarnitine species) and the ratios of metabolites (12 amino acid ratios and 18 ratios for acylcarnitines) were determined using LC-MS/MS and 2.5^th^, 50^th^ (median), and 97.5^th^ percentiles were calculated for three groups of healthy population (Supplementary File 1: Supplementary Table S5). For each analyte of the healthy populations, the upper limit cut-offs were set at above the 97.5^th^ percentiles, whereas the lower limit cut-offs were set at below the 2.5^th^ percentiles.  Table 1 presents the cut-off values for the analytes and marker ratios for screening of aminoacidopathies, organic acidemias, and FAO disorders. For screening of specific IEMs, we had also taken consideration of clinical manifestations of the patients, previous family histories, unexplained deaths of siblings, and consanguinity of the parents, etc.

### 3.3. Results of DBS Specimens from Clinically Suspected Patients Using LC-MS/MS

Among 273 clinically suspected patients with IEMs, 9 patients came out as screening positive for 6 different IEMs by LC-MS/MS analysis of DBS cards. Among these 9 patients, there were 3 cases of phenylketonuria (PKU); 1 case for each of citrullinemia type II (CIT-II), methylmalonic acidemia (MMA), isovaleric acidemia (IVA), and carnitine uptake defect (CUD); and 2 cases of medium-chain acyl-CoA dehydrogenase deficiency (MCAD).

Two female patients were found to be screening positive for PKU at the age of about 1 year and the only male patient was screening positive at the age of 10 years. Major clinical complications of these PKU patients were developmental delays, lethargy, and seizures (Table 2).

The male patient was positive for citrullinemia type II (CIT-II) upon screening at the age of 10 years and his major clinical complications included irritability, restlessness, and excessive crying followed by unconsciousness (Table 2).

A one-year-old boy with developmental delay, seizure, and low muscle tone was found to be screening positive for methylmalonic acidemia (MMA). A male patient who came out as screening positive for isovaleric acidemia (IVA) was diagnosed at the age of 1.9 years. He was hospitalized multiple times with clinical manifestations like acute respiratory infections and acute watery diarrhea. He had developmental delay and elevated level of plasma ammonia.

One male patient who was screening positive for carnitine uptake deficiency (CUD) was diagnosed at the age of 2 years had clinical complications including lethargy, restlessness, poor feeding, seizure, vomiting, abnormal movement, developmental delay, speech problems, and inability of walking. Two patients, a boy and a girl who were screening positive for medium-chain acyl-CoA dehydrogenase deficiency (MCAD), were siblings. The boy was 10 years of age, whereas the girl was aged 8.5 years at the time of screening. Common clinical complications of these patients were restlessness, irritability, abnormal behavior, speech problem, and mental retardation. The blood concentrations of C6, C8, C10, C10:1, and C8/C10 ratio of both the male and the female MCAD patients were slightly higher than the reference cut-off values, while the concentration of C10:2 and the C8/C10 ratio were within the reference cut-off values in case of both male and female patients (Table 2).

### 3.4. GC-MS-Based Second-Tier Test for Analysis of Urine Specimens from the LC-MS/MS-Based Screening Positive IEMs Cases

As mentioned earlier, nine patients came out as screening positive by LC-MS/MS analysis. Next, urine specimens from all these 9 patients were subjected to a second-tier test using GC-MS. The urinary metabolic profiling of 3 screening positive patients with PKU revealed elevated levels of 4-hydroxyphenylacetic acid, phenyllactic acid, 4-hydroxyphenyllactic acid, 2 hydroxyphenylacetic acid and mandelic acid. Elevated levels of these organic acids are suggestive of PKU caused by the deficiency of phenylalanine hydroxylase enzyme (Supplementary File 1: Supplementary Table S6).

In case of CIT-II screening positive patient, urinary metabolite profiling using GC-MS revealed an elevated level of citrulline, which is suggestive of citrullinemia (Supplementary File 1: Supplementary Table S6). GC-MS analysis of urine specimens from screening positive patient with MMA showed an elevated level of urinary excretion of methylmalonic acid, suggesting confirmatory diagnosis of the screening positive MMA case (Supplementary File 1: Supplementary Table S6). The second-tier test using GC-MS analysis of urine specimen from IVA screening positive case revealed that IVA specific metabolites such as isovalerylglycine and 3-OH-isovalerate were elevated (Supplementary File 1: Supplementary Table S6).

The urine metabolic profiling of screening positive patient with CUD revealed elevated levels of urinary excretions of 3-hydroxy butyric acid (3HB), adipic acid, and p-Cresol. An elevated level of adipic acid along with a highly elevated level of 3HB is suggestive of carnitine uptake deficiency (Supplementary File 1: Supplementary Table S6).

For the MCAD screening positive patients which had LC-MS/MS-derived borderline positive values above the cut-off, urinary metabolic profiling by GC-MS revealed that the levels of all the analytes tested were within the range of the cut-off, which confirms that these patients were not true cases of MCAD, i.e., false positive (Supplementary File 1: Supplementary Table S6).

## 4. Discussion

The number, intricacy, and diverse clinical spectrum of IEMs present a daunting diagnostic challenge to the physicians. However, in order to reduce morbidity and mortality, or other severe repercussions like irreversible neurological damage to the patients with IEMs, early diagnosis and institution of appropriate therapy are very critical. The use of LC-MS/MS during the past decades has led to a remarkable increase in screening of IEMs. Many countries have established newborn screening (NBS) tests using LC-MS/MS which analyzes metabolites from dried blood spots (DBSs) to detect the IEM-associated disorders, particularly the treatable one [30–32]. Although NBS is not in practice in Bangladesh, the government health policymakers have initiated official processes to start nationwide NBS program for management of IEMs. Under the circumstances, establishment of local cut-offs for IEMs-associated metabolites is timely. This is the first study on screening of IEMs using LC-MS/MS in Bangladesh, where the cut-off values of individual amino acids and acylcarnitines were established by analyzing the DBS specimens of healthy subjects and the patients with suspected IEMs were screened using the established cut-off values.

LC-MS/MS has been widely adopted for IEMs screening as it offers simultaneous and robust multiple disease screening using a single analytical high throughput technique [33]. Moreover, LC-MS/MS-based screening of IEMs provide the advantages of rapidity and convenience in sample collection and the stable isotopic internal standards used for quantification in this method increases the specificity and sensitivity of the test. In addition, it is possible to detect a number of common to very rare diseases inexpensively. To reduce the cost, usually a large number of specimens are analyzed in a single run.

Most aminoacidopathies, organic acidemias, and FAO disorders can be diagnosed using LC-MS/MS with 99% sensitivity and 99.995% specificity [1, 34–36] but it requires establishment of rigorous reference cut-off limits to detect the IEM-related disorders. Reliable cut-offs would help to minimize the false positive or false negative cases [14, 16, 37]. Through a worldwide collaborative project, the cut-off values for screening of IEMs were determined using 25-30 million healthy newborns, where 10742 cases were diagnosed with IEMs [38]. However, the cut-off limits of metabolites depend on different factors, such as analysis method, instrument platform, genetic background or ethnicity of a particular population [14, 39]. Since an NBS program does not yet exist in Bangladesh, the patients are hospitalized or they visit the physicians with clinical manifestations of IEMs during postneonatal period when irreversible damage has already occurred. Also, due to perplexing clinical presentation of IEMs signs and symptoms and lack of proper screening facilities, specimens are being sent to other countries where the results are interpreted by comparing the data with the cut-off values set for other geographical location and ethnic backgrounds. This study is an initiative to overcome those issues, as here, we have demonstrated the establishment of cut-off values for different amino acids and acylcarnitines for 3 different age groups. Sex-dependent variations of blood amino acids and acylcarnitines concentrations are very rare and this is why we generated only age-specific data considering age-specific variations of blood metabolites in healthy population are frequently reported [7, 17, 18, 40]. Moreover, the cut-off limits for screening of some amino acids and acylcarnitines are very close to normal reference intervals [18]. In the present study, all 7 patients who had been detected with IEMs were older than one year and we could successfully diagnose these patients using the established cut-off values for group B (8 days-7 years) and group C (8-17 years), which further emphasizes the necessity of age-specific cut-off ranges of different analytes.

In the study, among 273 patients with clinical signs and symptoms of IEMs, 7 (2.6%) came out as screening positive when DBS specimens were analyzed using LC-MS/MS. Different research groups across the world reported different frequencies of IEMs; e.g., Han and coworkers confirmed 1135 (6.2%) cases with IEMs among 18303 suspected inherited metabolic diseases in China [2], while Al Riyami S* et al. *reported a rate of 10.8% IEMs from Oman [41]. Furthermore, frequencies of 0.29%, 0.92% and 6% IEMs cases were reported from Korea, Turkey and Egypt, respectively. Our results are not directly comparable with other published survey data because the prevalence rates of IEMs vary due to geographical location, ethnicity, instrumentation platform, diagnostic strategies and time span of the surveillance in a particular population [13]. However, despite the difference in criteria for selection of IEMs-suspected patients and diagnostic approach, the results of our study are comparable with the published data from India, the neighboring country of Bangladesh, where IEM frequency has been reported to be as high as 3.2% (113 cases among 3550 suspected patients) [10]. Although the results of our study, which were generated in a time span of two years, do not reflect the true prevalence of IEMs in Bangladesh, it clearly demonstrates that IEMs are not uncommon in this country and thus the authorities in health sector and policymakers should be notified of the importance of screening of IEMs. In addition, consanguineous marriage, which is a common practice in Muslim countries like Bangladesh, is a major factor behind high rates of IEMs [42, 43]. We found that about 43% of our confirmed IEM cases came with consanguineous family history and the findings are consistent with others reports published across the globe.

Among 7 patients who had been detected with IEMs using the established cut-off ranges in this study, 3 had PKU (aminoacidopathies) and 1 patient for each of isovaleric acidemia and methylmalonic acidemia (organic academia), carnitine uptake deficiency and citrullinemia (urea cycle disorder). Our study identified aminoacidopathies with higher frequencies than other types of IEMs, and a similar pattern was also reported from two other neighboring countries, namely China and India, with PKU as the most prevalent IEM [10, 44]. All these 7 patients could possess a satisfactory quality of life provided that they would have undergone NBS and started receiving treatment earlier, because the IEM disorders they were suffering from were preventable. Thus, the effort to thwart disease progression and severity, and to provide diagnosed children with a tolerable living standard is only conceivable with the initiation and application of a nationwide screening program for IEMs in the first few days of life, followed by quick and specific treatment and care. Moreover, the benefits of such a program includes societal, ethical, and economic aspects, as the present health expenditures on handicapped people in Bangladesh are huge and beyond affordable limit for most people. Furthermore, the LC-MS/MS technology has been cost-effective for NBS in many developed countries as well as in developing countries [45–47]. Bangladesh's health policymakers must therefore consider LC-MS/MS technology for IEMs screening, at least for the most common disorders.

For the diagnosis of IEMs, the interpretation of LC-MS/MS results may be inconvenient or arguable if there is debate about the appropriateness of the reference ranges. In our study there were two false positive MCAD cases who had borderline positive concentrations of C6 acylcarnitines on LC-MS/MS analysis and these cases came out as negative when urinary GC-MS analysis was done. The reason for such false positive cases might be due to relatively low number of sample size for determination of cut-offs and this might be seen as the shortfall of the study. Analysis of more samples in the future is expected to generate more reliable cut-offs. In addition to screening, the second-tier tests need to be performed by experienced biochemical and genetic experts. Usually, various biochemical test, GC-MS, HPLC, enzymatic assay and molecular analysis can be used as a second-tier or confirmatory test. However, none of these tests offers the conveniences of LC-MS/MS which is why LC-MS/MS has been widely granted and used in NBS program all across the globe.

Finally, apart from establishing cut-off values for various amino acids and acylcarnitines for Bangladeshi population and screening for metabolic disorders in Bangladesh, the current study also aimed to gain practical experience in using the technology and evaluating the overall efficiency of the method in a Bangladeshi setting. We hope this experience would assist other researchers or government authorities in installing and establishing the new technology for screening of IEMs through NBS program.

In conclusion, LC-MS/MS techniques may play a vital role in screening and diagnosis of IEMs in newborns and this may be helpful in facilitating timely therapy of treatable IEMs. Furthermore, diagnosis of the relatively prevalent metabolic disorders among Bangladeshi population, along with their clinical features and ages of onsets, may provide physicians with a deeper understanding of these conditions, which would allow for early diagnosis and better treatment. Lastly, given the high birth rate and economical condition in countries like Bangladesh, introducing NBS for these disorders would be a considerable but worthwhile challenge.

## Figures and Tables

**Table 1:** The cut-off values of the analytes and marker ratios for screening of IEMs.

**Suspected disease**	**Analytes/Marker ratios**	**Cut-offs** **Group A** **(**μ**mol/L)**	**Cut-offs** **Group B** **(**μ**mol/L)**	**Cut-offs** **Group C** **(**μ**mol/L)**
**PKU**	↑ Phe	> 85.45	> 88.12	> 82.08
	↑ Phe/tyr	> 3.47	> 5.11	> 8.03

**BHD**	↑ Phe/tyr	> 3.47	> 5.11	> 8.03

**CIT**	↑ Cit	> 17.17	> 35.91	> 39.74
	↑ Cit/Phe	> 0.42	> 0.75	> 0.80

**TYR**	↑ Tyr	> 199.96	> 128.12	> 136.63
	↑ Tyr/Phe	> 5.90	> 2.50	> 2.56

**MSUD**	↑ Val	> 188.83	> 187.45	> 210.34
	↑ Xle	> 200.85	> 201.74	> 220.69
	↑ Val/Phe	> 5.37	> 3.35	> 4.05
	↑ Xle/Ala	> 1.37	> 1.44	> 1.23
	↑ Xle/Phe	> 5.62	> 3.37	> 4.14

**ARG**	↑ Arg	> 21.39	> 74.98	> 95.36
	↑ Arg/Orn	> 0.26	> 1.00	> 1.04

**OTC**	↓ Cit	< 4.68	< 11.51	< 13.67
	↑ Orn	> 112.77	> 145.61	> 148.49

**HCY**	↑ Met	> 42.38	> 40.65	> 42.17
	↑ Met/Phe	> 0.74	> 0.77	> 0.78

**MMA**	↑ C3	> 6.88	> 2.69	> 2.70
	↑ C3/C2	> 0.19	> 0.15	> 0.14

**IVA**	↑ C5	> 0.55	> 0.26	> 0.29
	↑ C5/C3	> 0.42	> 0.16	> 0.19

**GA-II**	↑ C4	> 0.63	> 0.33	> 0.38
	↑ C8	> 0.14	> 0.14	> 0.30
	↑ C14	> 0.51	> 0.27	> 0.13
	↑ C16	> 7.23	> 2.47	> 2.20
	↑ C12	> 0.23	> 0.14	> 0.15

**GA-I**	↑ C5DC	> 0.29	> 0.17	> 0.20

**CUD**	↓ C0	< 15.60	< 12.34	< 13.38
	↓ C2	< 6.88	< 8.71	< 9.05

**CPT-I**	↑ C0	> 73.73	> 69.76	> 75.67
	↑ C0/(C16+C18)	> 21.67	> 43.54	> 56.08
	↓ C16	< 0.88	< 0.61	< 0.55

**SCAD**	↑ C4	> 0.63	> 0.33	> 0.38
	↑ C4/C2	> 0.03	> 0.02	> 0.02

**MCAD**	↑ C6	> 0.09	> 0.09	> 0.12
	↑ C8	> 0.14	> 0.14	> 0.30
	↑ C10	> 0.18	> 0.23	> 0.58
	↑ C10:1	> 0.11	> 0.16	> 0.21
	↑ C10:2	> 0.02	> 0.08	> 0.08
	↑ C8/C10	> 1.67	> 4.43	> 2.45
	↑ C8/C2	> 0.01	> 0.01	> 0.02

**VLCAD**	↑ C14:1	> 0.34	> 0.16	> 0.26
	↑ C14:1/C16	> 0.09	> 0.16	> 0.22
	↑ C14	> 0.51	> 0.27	> 0.13

**CPT-II**	↑ C16	> 7.23	> 2.47	> 2.20

**CACT**	↑ C18	> 1.69	> 0.91	> 0.88
	↑ C18:1	> 1.98	> 1.57	> 1.67

**TFP**	↑ C18OH	> 0.05	> 0.01	> 0.01
	↑ C18:1OH	> 0.03	> 0.02	> 0.02

PKU, phenylketonuria; BHD, BH4 deficiency; CIT, citrullinemia; TYR, tyrosinemia; MSUD, maple syrup urine disease; ARG, argininemia; OTC, ornithine transcarbamylase deficiency; HCY, homocystinuria; MMA, methylmalonic acidemia; IVA, isovaleric acidemia; GA-II, glutaric acidemia type II (multiple acyl-CoA dehydrogenase deficiency); GA-I, glutaric acidemia type I; CUD, carnitine uptake defect; CPT-I, carnitine palmitoyltransferase I deficiency; SCAD, short-chain acyl-CoA dehydrogenase deficiency; MCAD, medium-chain acyl-CoA dehydrogenase deficiency; VLCAD, very long-chain acyl-CoA dehydrogenase deficiency; CPT-II, carnitine palmitoyltransferase II deficiency; CACT, carnitine-acylcarnitine translocase deficiency; TFP, trifunctional protein deficiency.

**Table 2:** Abnormal blood concentrations of metabolites and marker ratios together with clinical manifestations of the patients with suspected IEMs, as screened by LC-MS/MS.

**Name of disorders**	**Total no. of positive cases (frequency among detected cases)**	**Case ID**	**Age at diagnosis**	**Metabolites or marker ratios**	**Concentrations of metabolites (**μ**mol/L) or marker ratios**	**Cut-offs** **(**μ**mol/L)**	**Major clinical complications**
**PKU**	3 (33.3%)	Case 1	1.1 years	Phe	180.12	> 88.12	Lethargy, irritation, seizure and developmental delay
				Phe/tyr	5.97	> 5.11	
		Case 2	1.0 year	Phe	282.45	> 88.12	
				Phe/tyr	6.30	> 5.11	
		Case 3	10.0 years	Phe	1170.32	> 82.08	
				Phe/tyr	46.58	> 8.03	

**CIT-II**	1 (11.1%)	Case 4	10.0 years	Cit	1494.66	> 39.74	Irritability, restlessness and excessive crying followed by unconsciousness
				Cit/Phe	26.68	> 0.80	

**MMA**	1 (11.1%)	Case 5	1.0 year	C3	5.39	> 2.69	Developmental delay, seizure and low muscle tone
				C3/C2	0.29	> 0.15	

**IVA**	1 (11.1%)	Case 6	1.9 years	C5	13.80	> 0.26	Recurrent infection and developmental delay
				C5/C3	25.07	> 0.16	

**CUD**	1 (11.1%)	Case 7	2.0 years	C0	6.13	< 12.34	Lethargy, restless, poor feeding, seizure, vomiting, developmental delay, speech problem and inability of walking
				C2	6.47	< 8.71	

**MCAD**	2 (22.2%)	Case 8	10.0 years	C6	0.62	> 0.12	Restlessness, irritability, abnormal behavior, speech problem and mental retardation
				C8	1.90	> 0.30	
				C10	2.90	> 0.58	
				C10:1	1.14	> 0.21	
				C10:2	0.04	> 0.08	
				C8/C10	0.65	> 2.45	
				C8/C2	0.16	> 0.02	
		Case 9	8.5 years	C6	0.23	> 0.12	
				C8	1.15	> 0.30	
				C10	1.88	> 0.58	
				C10:1	0.66	> 0.21	
				C10:2	0.03	> 0.08	
				C8/C10	0.61	> 2.45	
				C8/C2	0.09	> 0.02	

PKU, phenylketonuria; CIT-II, citrullinemia type II; MMA, methylmalonic acidemia; IVA, isovaleric acidemia; CUD, carnitine uptake defect; and MCAD, medium-chain acyl-CoA dehydrogenase deficiency.

## Data Availability

All relevant data generated or analyzed during this study are included in this article and supporting information file.

## Supplementary Materials

Supplementary Materials Supplementary Table S1: Data acquisition parameters of MRM method for analysis of amino acids and acylcarnitines. Supplementary Table S2: Intra-assay precision and accuracy analysis of LC-MS/MS method for amino acids and acylcarnitines using low, medium and high control specimens. Supplementary Table S3: Inter-assay precision and accuracy analysis of LC-MS/MS method for amino acids, free carnitine, and acylcarnitines using low, medium, and high control specimens. Supplementary Table S4: Linearity, limit of detection (LOD), limit of quantitation (LOQ), and recovery analysis of LC-MS/MS method for amino acids and acylcarnitines using low, medium, and high control specimens. Supplementary Table S5: Percentile distribution of amino acids, acylcarnitines, and related ratios in different age groups of healthy participants. Supplementary Table S6: GC-MS-based urinary metabolic profiling for confirmatory diagnosis of the screening positive patients with IEMs.
