# Supplementary material for: Age-Specific Cut-off Values of Amino Acids and Acylcarnitines for Diagnosis of Inborn Errors of Metabolism Using Liquid Chromatography Tandem Mass Spectrometry
Source: Biomed Res Int. 2019 Jan 6;2019:3460902. doi: 10.1155/2019/3460902 (PMC6339774; doi:10.1155/2019/3460902)
Supplement: Supplementary Materials — Supplementary Table S1: Data acquisition parameters of MRM method for analysis of amino acids and acylcarnitines. Supplementary Table S2: Intra-assay precision and accuracy analysis of LC-MS/MS method for amino acids and acylcarnitines using low, medium and high control specimens. Supplementary Table S3: Inter-assay precision and accuracy analysis of LC-MS/MS method for amino acids, free carnitine, and acylcarnitines using low, medium, and high control specimens. Supplementary Table S4: Linearity, limit of detection (LOD), limit of quantitation (LOQ), and recovery analysis of LC-MS/MS method for amino acids and acylcarnitines using low, medium, and high control specimens. Supplementary Table S5: Percentile distribution of amino acids, acylcarnitines, and related ratios in different age groups of healthy participants. Supplementary Table S6: GC-MS-based urinary metabolic profiling for confirmatory diagnosis of the screening positive patients with IEMs. [file 3460902.f1.pdf]

Supplementary Table S1: Data acquisition parameters of MRM method for analysis of amino acids and acylcarnitines.

Analytes	MRM (Analyte)	Internal Standard	MRM (Internal Standard)	Target Dwell Time (msec)	Target Q1 Pre Bias (V)	Target Collision Energy (V)	Target Q3 Pre Bias (V)
**Ala**	90.1→44.2	$^{2}H_{4}$-Ala	94.1→48.2	5	-11	-13	-19
**Arg**	175.1→70.1	$^{2}H_{4}$-^{13}C-Arg	180.1→75.1	15	-13	-24	-29
**Cit**	176.1→70.1	$^{2}H_{2}$-Cit	178.1→72.1	5	-13	-25	-28
**Xle**	132.1→86.1	$^{2}H_{3}$-Leu	135.1→89.1	5	-10	-12	-16
**Lys**	147.1→84.1	$^{13}C_{6}$-$^{15}N_{2}$-Lys	155.1→90.1	10	-11	-18	-16
**Met**	150.2→61.2	$^{2}H_{3}$-Met	153.2→64.2	5	-11	-25	-26
**Orn**	133.2→70.2	$^{2}H_{6}$-Orn	139.2→76.2	10	-10	-23	-29
**Phe**	166.1→120.1	$^{13}C_{6}$-Phe	172.1→126.1	5	-12	-16	-12
**Pro**	116.2→70.2	$^{2}H_{5}$-Pro	121.2→74.2	5	-14	-18	-28
**Ser**	106.1→60.1	$^{13}C_{3}$-Ser	109.1→62.1	5	-12	-14	-24
**Tyr**	182.1→91.1	$^{13}C_{6}$-Tyr	188.1→142.1	5	-13	-15	-27
**Val**	118.1→72.2	$^{2}H_{8}$-Val	126.1→80.2	5	-14	-14	-14
**C0**	162.1→85	$^{2}H_{9}$-C0	171.1→85	5	-12	-23	-15
**C2**	204.1→85	$^{2}H_{3}$-C2	207.1→85	5	-10	-22	-14
**C3**	218.1→85	$^{2}H_{3}$-C3	221.1→85	5	-11	-23	-15
**C4**	232.2→85	$^{2}H_{3}$-C4	235.2→85	5	-12	-23	-15
**C5**	246.2→85	$^{2}H_{9}$-C5	255.2→85	5	-13	-23	-15
**C5:1**	244.2→85	$^{2}H_{9}$-C5	255.2→85	5	-13	-23	-15
**C5DC**	276.2→85	$^{2}H_{3}$-C5DC	279.2→85	10	-13	-25	-16

**C5OH**	262.2→85	$^{2}H_9$-C5	255.2→85	5	-13	-23	-15
**C6**	260.2→85	$^{2}H_3$-C6	263.2→85	5	-10	-24	-15
**C8**	288.2→85	$^{2}H_3$-C8	291.2→85	5	-11	-23	-15
**C10**	316.2→85	$^{2}H_3$-C10	319.2→85	5	-12	-25	-15
**C10:1**	314.2→85	$^{2}H_3$-C10	319.2→85	5	-12	-25	-15
**C10:2**	312.2→85	$^{2}H_3$-C10	319.2→85	5	-12	-25	-15
**C12**	344.2→85	$^{2}H_3$-C12	347.2→85	5	-13	-27	-14
**C14**	372.2→85	$^{2}H_9$-C14	381.2→85	5	-14	-28	-15
**C14:1**	370.2→85	$^{2}H_9$-C14	381.2→85	5	-14	-28	-15
**C14:2**	368.2→85	$^{2}H_9$-C14	381.2→85	5	-14	-28	-15
**C16**	400.3→85	$^{2}H_3$-C16	403.3→85	5	-15	-28	-15
**C16OH**	416.3→85	$^{2}H_3$-C16	403.3→85	5	-15	-28	-15
**C16:1OH**	414.3→85	$^{2}H_3$-C16	403.3→85	5	-15	-28	-15
**C18**	428.3→85	$^{2}H_3$-C18	431.3→85	5	-16	-30	-15
**C18:1**	426.3→85	$^{2}H_3$-C18	431.3→85	5	-16	-30	-15
**C18OH**	444.3→85	$^{2}H_3$-C18	431.3→85	5	-16	-30	-15
**C18:1OH**	442.3→85	$^{2}H_3$-C18	431.3→85	5	-16	-30	-15

Abbreviations: MRM, Multiple Reaction Monitoring; MRM transitions: precursor ion → target ion; Ala, alanine; Arg, arginine; Cit, citrulline; Xle, (Leucine and isoleucine); Lys, lysine; Met, methionine; Orn, ornithine; Phe, phenylalanine; Pro, proline; Ser, serine; Tyr, tyrosine; Val, valine; C0, free carnitine; C2, acetylcarnitine; C3, propionylcarnitine; C4 butyryl-/isobutyrylcarnitine; C5, isovaleryl-/2-methylbutyrylcarnitine, C5:1, tiglylcarnitine; C5DC, glutarylcarnitine; C5OH, hydroxy isovalerylcarnitine; C6, hexanoylcarnitine; C8, octanoylcarnitine; C10, decanoylcarnitine; C10:1, decenoylcarnitine; C10:2, decadienoylcarnitine; C12, dodecanoylcarnitine; C14, tetradecanoylcarnitine; C14:1, tetradecenoylcarnitine; C14:2, tetradecadienoylcarnitine; C16, palmitoylcarnitine; C16OH, hydroxy palmitoylcarnitine; C16:1OH, hydroxy hexadecenoylcarnitine; C18, stearylcarnitine; C18:1, oleylcarnitine; C18OH, hydroxy stearylcarnitine; C18:1OH, hydroxy oleylcarnitine.

Supplementary Table S2: Intra-assay precision and accuracy analysis of LC-MS/MS method for amino acids and acylcarnitines using low, medium and high control specimens.

Analytes	Low Control			Medium Control			High Control			**Average intra-assay CV (%)**	**Average RE (%)**
	Target (µmol/L)	Intra assay mean (µmol/L)	Intra assay CV (%)	Target (µmol/L)	Intra assay mean (µmol/L)	Intra assay CV (%)	Target (µmol/L)	Intra assay mean (µmol/L)	Intra assay CV (%)		
Ala	149..6	128.54	16.72	465.1	357.41	20.34	767	595.87	13.25	16.77	-19.85
Arg	38.7	32.2	5.46	100.6	102.87	2.43	374.6	467.33	0.95	2.95	3.41
Cit	34	30.78	14.82	77.5	75.669	9.84	282.6	322.15	12.01	12.22	0.73
Xle	160	162.61	7.34	483.4	493.84	7.17	767.2	814.62	4.75	6.42	3.32
Lys	80.8	71	4.63	123.9	121.37	3.89	332.9	353.93	2.66	3.73	-2.62
Met	35.7	38.54	12.37	117	110.95	9.08	506.6	475.56	5.47	8.97	-1.11
Orn	57	40.4	10.1	110	99.68	6.99	351.5	389.29	3.77	6.95	9.33
Phe	55.9	57.74	7.08	121.3	123.17	4.89	425	441	3.97	5.31	2.87
Pro	123.7	124.87	3.14	401.5	357.17	5.13	684.6	656.88	3.26	3.84	-4.72
Ser	168.6	153.2	23.68	533.1	566.67	16.07	892.8	897.62	9.03	16.26	-0.77
Tyr	106.4	115.37	33.8	283.2	280.34	12.7	501.5	440.17	22.94	23.15	-1.6
Val	88.1	88.37	11.69	182.4	173.55	9.22	589.5	557.49	4.44	8.45	-3.33
C0	20.57	20.96	12.82	49.5	51.39	6.8	90.6	94.45	5.24	8.29	3.32
C2	24.97	23.63	2.92	67.5	69.66	4.42	127.7	137.27	4.86	4.07	1.77
C3	3.6	3.65	3.79	11.2	11.62	6.4	21.8	22.43	3.82	4.67	2.72
C4	0.16	0.15	9.91	0.76	0.79	4.18	1.5	1.67	5.21	6.43	2.21
C5	0.25	0.23	15.29	1.49	1.64	8.26	3.06	3.57	5.1	9.55	6.93
C6	0.08	0.08	37.06	0.65	0.67	3.61	1.35	1.46	5.73	15.47	3.93
C8	0.13	0.1	90.58	1.01	1.18	10.47	2.12	2.55	6.15	35.73	5.03
C10	0.06	0.08	25.58	0.46	0.46	5.31	0.96	0.94	5.7	12.20	9.00
C12	0.06	0.07	21.11	0.43	0.42	6.71	0.91	0.83	5.03	10.95	1.81
C14	0.12	0.11	8.43	0.45	0.43	7.25	0.87	0.82	4.44	6.71	-6.46
C16	3.85	3.78	1.93	9.24	9.76	2.63	16.67	17.76	1.49	2.02	3.45
C18	0.97	0.98	3.42	1.93	2.06	2.8	3.32	3.49	4.06	3.43	4.29

Abbreviations: RE, relative error; Ala, alanine; Arg, arginine; Cit, citrulline; Xle, (Leucine and isoleucine); Lys, lysine; Met, methionine; Orn, ornithine; Phe, phenylalanin; Pro, proline; Ser, serine; Tyr, tyrosine; Val, valine; C0, free carnitine; C2, acetylcarnitine; C3, propionylcarnitine; C4 butyryl-/isobutyrylcarnitine; C5, isovaleryl-/2-methylbutyrylcarnitine; C6, hexanoylcarnitine; C8, octanoylcarnitine; C10, decanoylcarnitine; C12, dodecanoylcarnitine; C14, tetradecanoylcarnitine; C16, palmitoylcarnitine and C18, stearylcarnitine.

Supplementary Table S3: Inter-assay precision and accuracy analysis of LC-MS/MS method for amino acids, free carnitine and acylcarnitines using low, medium and high control specimens.

Analytes	Low Control			Medium Control			High Control			Average intra-assay CV (%)	Average RE (%)
	**Target (µmol/L)**	**Inter assay mean (µmol/L)**	**Inter assay CV (%)**	**Target (µmol/L)**	**Inter assay mean (µmol/L)**	**Inter assay CV (%)**	**Target (µmol/L)**	**Inter assay mean (µmol/L)**	**Inter assay CV (%)**		
Ala	149.6	129.56	6.85	465.1	361.5	14.4	767	596.23	2.3	7.85	-19.31
Arg	38.7	32.37	4.64	100.6	102.86	0.85	374.6	467.37	0.42	1.97	3.55
Cit	34	30.93	7.31	77.5	76.15	5.74	282.6	322.81	1.78	4.94	1.15
Xle	160	163.23	4.63	483.4	492.23	3.71	767.2	812.81	1.98	3.44	3.26
Lys	80.8	71.12	1.58	123.9	121.1	1.94	332.9	353.81	0.45	1.32	-2.65
Met	35.7	38.51	3.38	117	111.28	2.57	506.6	475.17	3.97	3.31	-1.07
Orn	57	40.67	6.2	110	99.88	2.16	351.5	390.43	2.52	3.63	-9
Phe	55.9	57.53	4.31	121.3	122.54	5.17	425	442.09	2.35	3.94	2.65
Pro	123.7	124.71	1.59	401.5	357.15	4	684.6	657.23	1.53	2.37	-4.74
Ser	168.6	156.31	18.21	533.1	571.77	10.1	892.8	894.98	6.48	11.60	0.07
Tyr	106.4	117.31	15.05	283.2	281.14	7.13	501.5	436.47	8	10.06	-1.15
Val	88.1	88.29	2.36	182.4	173.89	7.49	589.5	556.5	2.71	4.19	-3.56
C0	20.57	21.04	6.36	49.5	51.6	3.93	90.6	94.36	1.06	3.78	3.56
C2	24.97	23.64	1.19	67.5	69.58	1.75	127.7	137.58	1.97	1.64	1.83
C3	3.6	3.65	1.08	11.2	11.62	5.14	21.8	22.42	0.88	2.37	2.67
C4	0.16	0.14	9.17	0.76	0.79	2.17	1.5	1.67	2.05	4.46	1.83
C5	0.25	0.23	8.64	1.49	1.64	1.74	3.06	3.58	2.98	4.45	6.66
C6	0.08	0.08	25.33	0.65	0.67	1	1.35	1.46	0.41	8.91	3.45
C8	0.13	0.1	27.9	1.01	1.17	11.05	2.12	2.56	3.47	14.14	4.82
C10	0.06	0.08	24.51	0.46	0.46	3.7	0.96	0.94	4.21	10.81	8.36
C12	0.06	0.07	12.59	0.43	0.42	1.74	0.91	0.83	4.15	6.16	2.19
C14	0.12	0.11	2.93	0.45	0.43	5.02	0.87	0.82	2.53	3.49	-6.61
C16	3.85	3.78	1.25	9.24	9.74	1.69	16.67	17.7	0.53	1.16	3.38
C18	0.97	0.98	2..53	1.93	2.06	0.51	3.32	3.48	3.24	1.88	4.08

Abbreviations: RE, relative error; Ala, alanine; Arg, arginine; Cit, citrulline; Xle, (Leucine and isoleucine); Lys, lysine; Met, methionine; Orn, ornithine; Phe, phenylalanin; Pro, proline; Ser, serine; Tyr, tyrosine; Val, valine; C0, free carnitine; C2, acetylcarnitine; C3, propionylcarnitine; C4 butyryl-/isobutyrylcarnitine; C5, isovaleryl-/2-methylbutyrylcarnitine; C6, hexanoylcarnitine; C8, octanoylcarnitine; C10, decanoylcarnitine; C12, dodecanoylcarnitine; C14, tetradecanoylcarnitine; C16, palmitoylcarnitine and C18, stearylcarnitine.

Supplementary Table S4: Linearity, Limit of detection (LOD), Limit of quantitation (LOQ) and recovery analysis of LC-MS/MS method for amino acids and acylcarnitines using low, medium and high control specimens.

Analytes	R^2	y-Intercept	Slope	LOD	LOQ	Recovery (%)
Ala	0.9997	14.38	0.76	48.53	147.05	80.68
Arg	0.9995	-22.80	1.31	28.91	87.62	103.54
Cit	0.9996	-12.12	1.18	19.61	59.42	101.15
Leu	0.9991	-13.02	1.07	91.65	277.72	103.26
Lys	1.0000	-18.48	1.12	7.10	21.51	97.34
Met	1.0000	4.07	0.93	10.40	31.50	98.92
Orn	0.9999	-29.09	1.19	13.29	40.27	90.99
Phe	0.9999	-2.40	1.05	12.09	36.64	102.65
Pro	0.9954	-3.31	0.95	179.85	545.01	95.25
Ser	0.9954	-3.31	0.95	179.85	545.01	100.06
Tyr	0.9942	39.47	0.80	147.06	445.65	98.85
Val	1.0000	4.69	0.94	11.02	33.38	96.65
C0	1.0000	-0.38	1.05	1.47	4.45	103.55
C2	0.9998	-4.56	1.11	5.97	18.08	101.83
C3	0.9999	-0.01	1.03	0.66	1.99	102.66
C4	0.9993	-0.06	1.14	0.15	0.44	101.82
C5	0.9995	-0.09	1.19	0.26	0.80	106.65
C6	0.9995	-0.02	1.09	0.12	0.37	103.44
C8	0.9999	-0.07	1.24	0.06	0.20	104.82
C10	1.0000	0.02	0.96	0.01	0.03	108.35
C12	0.9994	0.02	0.89	0.10	0.29	102.18
C14	0.9997	0.00	0.95	0.04	0.12	93.37
C16	0.9999	-0.36	1.08	0.57	1.72	103.37
C18	0.9992	-0.03	1.06	0.35	1.07	104.08

Abbreviations: Ala, alanine; Arg, arginine; Cit, citrulline; Xle, (Leucine and isoleucine); Lys, lysine; Met, methionine; Orn, ornithine; Phe, phenylalanin; Pro, proline; Ser, serine; Tyr, tyrosine; Val, valine; C0, free carnitine; C2, acetylcarnitine; C3, propionylcarnitine; C4 butyryl-/isobutyrylcarnitine; C5, isovaleryl-/2-methylbutyrylcarnitine; C6, hexanoylcarnitine; C8, octanoylcarnitine; C10, decanoylcarnitine; C12, dodecanoylcarnitine; C14, tetradecanoylcarnitine; C16, palmitoylcarnitine and C18, stearylcarnitine; R^2, Coefficient of determination; LOD, Limit of detection and LOQ, Limit of quantitation

Supplementary Table S5: Percentile distribution of amino acids, acylcarnitines and related ratios in different age groups of healthy participants.

	Group A			Group B			Group C		
	Percentiles			Percentiles			Percentiles		
	2.50	50.00	97.50	2.50	50.00	97.50	2.50	50.00	97.50
**Amino acids**									
**Ala**	74.36	136.60	282.85	88.18	190.95	339.86	111.76	205.15	405.78
**Arg**	0.50	4.57	21.39	3.26	19.51	74.98	4.26	29.78	95.36
**Cit**	4.68	9.78	17.17	11.51	20.83	35.91	13.67	24.27	39.74
**Xle**	70.02	114.62	200.85	75.68	128.43	201.74	102.02	155.75	220.69
**Lys**	57.34	166.53	416.22	57.39	201.54	295.92	67.03	237.56	379.11
**Met**	7.51	17.96	42.38	10.61	22.45	40.65	11.69	22.03	42.17
**Orn**	21.29	55.87	112.77	34.98	86.88	145.61	51.48	94.93	148.49
**Phe**	27.17	42.54	85.45	32.34	52.40	88.12	33.54	53.72	82.08
**Pro**	84.25	145.36	320.14	76.18	136.11	256.02	92.59	158.86	324.41
**Ser**	186.92	339.78	581.31	111.44	198.15	374.39	123.99	209.80	370.87
**Tyr**	7.11	82.40	199.96	9.19	44.16	128.12	0.37	47.95	136.63
**Val**	51.55	101.89	188.83	52.78	117.78	187.45	59.58	138.84	210.34
**Amino acid ratio**									
**Phe/Tyr**	0.16	0.54	3.47	0.39	1.20	5.11	0.06	1.13	8.03
**Val/Phe**	1.13	2.37	5.37	1.12	2.11	3.35	1.24	2.52	4.05
**Leu/Ala**	0.37	0.80	1.37	0.34	0.66	1.44	0.35	0.74	1.23
**Leu/Phe**	1.43	2.51	5.62	1.53	2.48	3.37	1.83	2.75	4.14
**Met/Phe**	0.21	0.43	0.74	0.21	0.43	0.77	0.22	0.41	0.78
**Met/Leu**	0.05	0.17	0.39	0.09	0.18	0.29	0.08	0.15	0.26
**Met/Cit**	0.72	1.86	4.77	0.47	1.09	2.16	0.47	0.91	1.83
**Met/Tyr**	0.06	0.23	1.15	0.18	0.48	2.05	0.03	0.47	2.55
**Cit/Phe**	0.10	0.23	0.42	0.21	0.39	0.75	0.24	0.46	0.80
**Cit/Arg**	0.53	2.25	16.26	0.31	1.05	5.68	0.30	0.86	4.89
**Tyr/Phe**	0.13	1.82	5.90	0.19	0.83	2.50	0.01	0.86	2.56
**Arg/Orn**	0.01	0.08	0.26	0.05	0.21	1.00	0.05	0.36	1.04
**Acylcarnitines**									
**C0**	15.60	32.00	73.73	12.34	39.18	69.76	13.38	43.53	75.67
**C2**	6.88	20.39	64.68	8.71	16.57	34.90	9.05	15.63	29.31
**C3**	0.61	1.86	6.88	0.63	1.38	2.69	0.64	1.40	2.70
**C4**	0.08	0.18	0.63	0.06	0.13	0.33	0.05	0.15	0.38
**C5**	0.09	0.19	0.55	0.04	0.11	0.26	0.05	0.13	0.29
**C5:1**	0.00	0.00	0.02	0.00	0.00	0.02	0.00	0.01	0.02
**C5DC**	0.04	0.11	0.29	0.00	0.06	0.17	0.00	0.07	0.20
**C5OH**	0.08	0.14	0.25	0.12	0.25	0.45	0.13	0.27	0.53
**C6**	0.00	0.03	0.09	0.00	0.02	0.09	0.00	0.02	0.12

**C8**	0.00	0.02	0.14	0.00	0.03	0.14	0.00	0.04	0.30
**C10**	0.03	0.08	0.18	0.00	0.06	0.23	0.01	0.08	0.58
**C10:1**	0.02	0.05	0.11	0.00	0.05	0.16	0.00	0.07	0.21
**C10:2**	0.00	0.01	0.02	0.00	0.02	0.08	0.00	0.02	0.08
**C12**	0.03	0.08	0.23	0.00	0.05	0.14	0.00	0.04	0.15
**C14**	0.11	0.21	0.51	0.04	0.08	0.27	0.03	0.07	0.13
**C14:1**	0.04	0.11	0.34	0.03	0.07	0.16	0.03	0.08	0.26
**C14:2**	0.01	0.02	0.05	0.00	0.02	0.07	0.00	0.03	0.12
**C16**	0.88	3.07	7.23	0.61	1.19	2.47	0.55	1.15	2.20
**C16OH**	0.00	0.01	0.05	0.00	0.01	0.01	0.00	0.00	0.01
**C16:1OH**	0.01	0.03	0.07	0.00	0.02	0.08	0.00	0.02	0.05
**C18**	0.32	0.69	1.69	0.25	0.47	0.91	0.22	0.40	0.88
**C18:1**	0.45	0.94	1.98	0.46	0.82	1.57	0.42	0.79	1.67
**C18OH**	0.00	0.01	0.05	0.00	0.00	0.01	0.00	0.00	0.01
**C18:1OH**	0.01	0.02	0.03	0.00	0.01	0.02	0.00	0.01	0.02
**Acylcarnitines ratio**									
**C0/(C16+C18)**	3.75	8.45	21.67	7.12	23.57	43.54	7.96	27.07	56.08
**C3/C2**	0.04	0.09	0.19	0.04	0.08	0.15	0.05	0.08	0.14
**C3/C16**	0.22	0.64	1.42	0.50	1.12	2.37	0.49	1.23	2.36
**C3/Met**	0.03	0.10	0.33	0.02	0.06	0.17	0.03	0.06	0.15
**C4/C2**	0.00	0.01	0.03	0.00	0.01	0.02	0.00	0.01	0.02
**C4/C3**	0.04	0.11	0.28	0.04	0.09	0.27	0.05	0.10	0.27
**C5/C3**	0.04	0.10	0.42	0.03	0.08	0.16	0.04	0.09	0.19
**C5/C0**	0.00	0.01	0.01	0.00	0.00	0.01	0.00	0.00	0.01
**C5OH/C8**	0.00	2.18	165.14	0.00	4.38	72.55	0.00	4.04	87.13
**C5OH/C3**	0.02	0.08	0.24	0.07	0.18	0.40	0.08	0.20	0.43
**C5OH/C0**	0.00	0.00	0.01	0.00	0.01	0.02	0.00	0.01	0.02
**C8/C10**	0.00	0.27	1.67	0.00	0.53	4.43	0.00	0.48	2.45
**C8/C2**	0.00	0.00	0.01	0.00	0.00	0.01	0.00	0.00	0.02
**C5DC/C5OH**	0.28	0.72	1.66	0.00	0.22	0.80	0.00	0.24	1.06
**C14:1/C2**	0.00	0.01	0.02	0.00	0.00	0.01	0.00	0.01	0.01
**C14:1/C16**	0.02	0.04	0.09	0.02	0.06	0.16	0.02	0.07	0.22
**C16OH/C16**	0.00	0.00	0.01	0.00	0.00	0.01	0.00	0.00	0.01
**C18OH/C18**	0.00	0.02	0.04	0.00	0.01	0.03	0.00	0.01	0.04

Abbreviations: Ala, alanine; Arg, arginine; Cit, citrulline; Xle, (Leucine and isoleucine); Lys, lysine; Met, methionine; Orn, ornithine; Phe, phenylalanine; Pro, proline; Ser, serine; Tyr, tyrosine; Val, valine; C0, free carnitine; C2, acetylcarnitine; C3, propionylcarnitine; C4 butyryl-/isobutyrylcarnitine; C5, isovaleryl-/2-methylbutyrylcarnitine, C5:1, tiglylcarnitine; C5DC,

glutarylcarnitine; C5OH, hydroxy isovalerylcarnitine; C6, hexanoylcarnitine; C8, octanoylcarnitine; C10, decanoylcarnitine; C10:1, decenoylcarnitine; C10:2, decadienoylcarnitine; C12, dodecanoylcarnitine; C14, tetradecanoylcarnitine; C14:1, tetradecenoylcarnitine; C14:2, tetradecadienoylcarnitine; C16, palmitoylcarnitine; C16OH, hydroxy palmitoylcarnitine; C16:1OH, hydroxy hexadecenoylcarnitine; C18, stearylcarnitine; C18:1, oleylcarnitine; C18OH, hydroxy stearylcarnitine; C18:1OH, hydroxy oleylcarnitine.

Supplementary Table S6: GC-MS-based urinary metabolic profiling for confirmatory diagnosis of the screening-positive patients with IEMs.

**Name of disorder**	**Case No.**	**Metabolites detected**	**Concentration (µmol/mmol of creatinine)**	**Biological reference interval (µmol/mmol of creatinine)[a]**
**PKU**	Case 1	4-Hydroxyphenylacetic acid	392	3.2 – 180.2
		4-Hydroxyphenyllactic acid	31.6	0 – 8.7
	Case 2	2-Hydroxyphenylacetic acid	52.3	0 – 20
		Phenyllactic acid	11.9	0 – 4
		4-Hydroxyphenylacetic acid	343	3.2 – 180.2
		4-Hydroxyphenyllactic acid	24.9	0 – 8.7
		Mandelic acid	117	0 - 64
	Case 3	2-Hydroxyphenylacetic acid	94.7	0 – 20
		Phenyllactic acid	38.6	0- 4
		4-Hydroxyphenylacetic acid	278	3.2 – 180.2
		4-Hydroxyphenyllactic acid	32.4	0 – 8.7
		Mandelic acid	95	0 - 64
**CIT-II**	Case 4	Citrulline	18803	10 - 99
**MMA**	Case 5	Methylmalonic acid	10.4	0 - 6
**IVA**	Case 6	Isovalerylglycine	91.4	0 - 10
		3-OH-isovalerate	65.3	3.1 23.1
**CUD**	Case 7	3-Hydroxybutyric acid	108	0 -11.1
		3-Hydroxyisovaleric Acid	64.7	3.1 – 23.1
		Adipic acid	63.2	0 – 35
		p-Cresol	57.8	0 - 11
**MCAD**	Case 8	Hexanoyl glycine	0.0	0 – 2.9
		Octanoyl glycine	0.0	0 – 0.2
		Phenylpropionyl glycine	0. 0	0 – 0.1
		Adipic acid	0.1	0 - 35
		Octanoic acid	0.0	0 – 7.7
		Sebacic acid	0.0	0 – 12.3
	Case 9	Hexanoyl glycine,	0.0	0 – 2.9

		Octanoyl glycine,	0.0	0 – 0.2
		Phenylpropionyl glycine,	0.0	0 – 0.1
		Adipic acid	0.1	0 - 35
		Octanoic acid	0.0	0 – 7.7
		Sebacic acid	0.0	0 – 12.3

Abbreviations: PKU, phenylketonuria; CIT-II, citrullinemia type II; MMA, methylmalonic acidemia; IVA, isovaleric acidemia; CUD, carnitine uptake defect; and MCAD, medium-chain acyl-CoA dehydrogenase deficiency. [a]Reference ranges were obtained from Human Metabolome Database (Version 4.0)[1].

References

1. Wishart DS, Feunang YD, Marcu A, Guo AC, Liang K, Vázquez-Fresno R et al. HMDB 4.0: the human metabolome database for 2018. Nucleic acids research. 2017;46(D1):D608-D17.
